# Pursuing Connectivity in Cardio-Oncology Care—The Future of Telemedicine and Artificial Intelligence in Providing Equity and Access to Rural Communities

**DOI:** 10.3389/fcvm.2022.927769

**Published:** 2022-06-13

**Authors:** Coralea Kappel, Moira Rushton-Marovac, Darryl Leong, Susan Dent

**Affiliations:** ^1^Department of Medicine, McMaster University, Hamilton, ON, Canada; ^2^Division of Medical Oncology, The Ottawa Hospital Cancer Centre, University of Ottawa, Ottawa, ON, Canada; ^3^Department of Health Research Methods, Evidence and Impact, McMaster University, Hamilton, ON, Canada; ^4^The Population Health Research Institute, McMaster University and Hamilton Health Sciences, Hamilton, ON, Canada; ^5^Division of Medical Oncology, Duke Cancer Institute, Duke University, Durham, NC, United States

**Keywords:** cardio-oncology, telehealth, artificial intelligence, innovation, care delivery model

## Abstract

The aim of this review is to discuss the current health disparities in rural communities and to explore the potential role of telehealth and artificial intelligence in providing cardio-oncology care to underserviced communities. With advancements in early detection and cancer treatment, survivorship has increased. The interplay between cancer and cardiovascular disease, which are the leading causes of morbidity and mortality in this population, has been increasingly recognized. Worldwide, cardio-oncology clinics (COCs) have emerged to deliver a multidisciplinary approach to the care of patients with cancer to mitigate cardiovascular risks while minimizing interruptions in cancer treatment. Despite the value of COCs, the accessibility gap between urban and rural communities in both oncology and cardio-oncology contributes to health care disparities and may be an underrecognized determinant of health globally. Telehealth and artificial intelligence offer opportunities to provide timely care irrespective of rurality. We therefore explore current developments within this sphere and propose a novel model of care to address the disparity in urban vs. rural cardio-oncology using the experience in Canada, a geographically large country with many rural communities.

## Introduction

Cardiovascular disease (CVD) and cancer are leading causes of morbidity and mortality worldwide, collectively responsible for almost half of all deaths globally (1). As per GLOBOCAN 2020, there was an estimated 19.3 million new cancer cases and almost 10.0 million cancer deaths worldwide in 2020 (2). Nearly half of all Canadians develop cancer in their lifetime (3) with cancer and cardiovascular comorbidities and toxicities now representing the leading causes of morbidity and mortality in cancer survivors (4–6). Given the complexity of these patients' cardiovascular and cancer needs, a multidisciplinary approach is recommended (7).

In the last decade, the field of oncology has seen remarkable progress in the early detection and management of solid and hematological malignancies which has translated to substantial improvements in disease-free and overall survival (8, 9). Even in the setting of non-curable cancers, contemporary therapies often lead to long-term disease control, requiring management akin to other chronic diseases. Along with the success of novel cancer therapies, unique treatment related toxicities, including cardiovascular toxicity, have emerged which require prompt recognition and treatment by health care providers.

Cardio-oncology has emerged as a discipline that has an increasingly important role in the care of patients receiving cancer treatment. This includes baseline cardiovascular risk assessment, prevention, identification and treatment of cardiovascular toxicities during treatment, and management of long-term cardiovascular complications following completion of treatment (10). There is increasing evidence to support the benefit of providing cardio-oncology services to patients with cancer, including the ability to complete the prescribed cancer therapy safely (11).

Cardio-oncology clinics (COCs) have arisen worldwide and across Canada over the last decade; however, they have been limited to larger academic and urban centers. Health care systems need to develop innovative ways of delivering accessible and timely cardio-oncology care for patients with cancer in both rural and remote communities.

## Health Disparities in Rural Communities

Health care accessibility is a determinant of health and remains a major reason for the health care gap between rural and urban regions worldwide, including Canada (12, 13). Worldwide, 56% of people living in rural areas do not have access to essential health-care services, more than double those in urban areas (13). Individuals in rural counties also have an 8% higher overall cancer mortality than their urban counterparts (14). Canada, among other parts of the world, faces the challenge of providing high-quality specialty care to large rural areas with low population density. Canada is the second-largest country in the world, covering over 9.88 million km^2^ while its population density is approximately four people per square kilometers which is among the lowest in the world (15). Furthermore, almost one-fifth of the population lives in rural communities (16), in contrast only 3% of all specialists are located in these areas (17). Therefore, despite universal health insurance in Canada, access to care remains an important social determinant of health with discrepancies in access between rural and urban areas. Rurality has been identified as a type of vulnerability (18).

People living in rural areas face unique social and economic challenges compared to their urban counterparts (19), including but not limited to, inconvenience and cost of travel (20, 21), absenteeism from work and family (20) and dependence on caregivers for transport or childcare (22). Several studies have found that increased travel time affects patient-care decisions as it relates to their cancer treatment (18, 23, 24) and more specifically when travel for treatment was longer than 1 hour, there was an associated increase in unmet patient needs (25). In a longitudinal review of rural health policy in Ontario, other rural health challenges included lower population health status scores and difficulty in recruiting and retaining healthcare professionals (26). The cancer care gradient across Canada is an important population health determinant (27); rural residence negatively affects access to treatment and decisions regarding treatment plan (28). In a population-based retrospective study, rural residence was a factor associated with absence of a specialist consultation during cancer care (OR 0.48, 95% CI 0.48–0.72) (29). Most Indigenous people living in Canada reside in remote communities where their cancer rates are increasing and survival is worse for all cancers compared with the general Canadian population. There is a lack of longitudinal community and public health programs, and distance among other factors limits access to screening and diagnostic follow-up (27). On the other hand, receiving care in a patient's rural community may be a benefit in terms of coping with cancer and maintaining close relationships with their families and community members (30). In addition to cardio-oncology care, we recognize that there are gaps in access to basic cardiology and oncology services in rural communities. Our hope is that health care providers and organizations can use principles outlined in this paper to improve access to all aspects of health care for these individuals.

### Importance of Multidisciplinary Care *via* COCs

Specialized care in cardio-oncology has emerged over the last decade to improve cancer outcomes by optimizing cardiovascular (CV) risks and reducing interruptions in cancer treatment secondary to CV events to ultimately mitigate morbidity and mortality from CVD. Globally, there are 21 countries with national cardio-oncology programs as per the International Cardio-Oncology Society registry, however the majority are in urban centers. Furthermore, 81% (*n* = 17) of centers are in upper-middle to high-income countries and there are currently no COCs registered in low-income countries (31).

In a recent position statement by the European Society of Cardiology in Collaboration with the International Cardio-Oncology Society, withholding effective but potentially cardiotoxic cancer treatment in patients at high or very high risk of CVD should be made after a multidisciplinary team discussion to balance cancer treatment efficacy and safety (32). Cardinale et al. (33) demonstrated that if anthracycline-induced cardiotoxicity is detected early, medical intervention can reverse cardiac damage, thus supporting the importance of early referral and widely available cardio-oncology services. In a cohort study of 779 cancer patients referred to The Ottawa Hospital COC, most breast cancer patients with LV dysfunction were able to complete their cancer treatments as a result of the collaborative approach between oncologists and cardiologists facilitated through a COC (11). Therefore, access to this multidisciplinary team, including an oncologist, cardiologist, pharmacist, and other allied health care providers, is a critical aspect of cancer patients' care that should be available irrespective of geographical location.

### The Role of Telehealth to Address the Care Gaps in Rural Communities

Telehealth has been defined as a service delivery system that uses communication technologies to deliver specialized services in real-time across geographical distances (34). Telehealth has emerged as an effective approach to promote accessible health care in rural communities (35). For cancer patients, video consultation is both feasible and effective (36). There is also consistent evidence that telehealth has an overall positive impact on both patient and caregiver satisfaction, and enhances access to health care for those living in rural and remote areas (35).

Despite the advantages of telehealth, there are several challenges that influence its success and sustainability. These include government support, reimbursement capacity, adaptability to the targeted population, and efficient administration of clinical processes (37, 38). In reviewing telehealth in cancer care during COVID-19, patients aged ≥70 years old were identified as having less participation in telehealth compared to in-person care. Video technology or internet may be less accessible in this age group (22). Some telehealth models have integrated a nurse or telehealth technician onsite to assist with appointment scheduling, technology troubleshooting and clinical examination (35). Although a pulmonary telehealth study found no negative effect on physicians' decision-making process, remote examination *via* a surrogate provider could affect the diagnostic process (39). Furthermore, virtual visits are limited by the inability of health care providers to perform the traditional physical exam, which may in some instances provide additional information. Another limitation is poor broadband internet access in rural regions, among racial and ethnic minorities, older adults, and in those with lower levels of education and income (40). Universal access to broadband internet is a determinant of access to telemedicine and should be a priority for policymakers (41). There are also financial limitations with respect to cost of equipment (e.g., automated blood pressure cuffs, oxygen saturation monitors, wireless ECG monitors such as KardiaMobile), and inability to obtain some investigations rurally such as echocardiograms.

Echocardiogram surveillance for left ventricular dysfunction is currently recommended in individuals receiving HER2-targeted therapies and anthracyclines; several other cancer therapies such as immune checkpoint inhibitors, certain tyrosine kinase inhibitors, and cyclophosphamide, have been associated with heart failure and it may be reasonable to consider surveillance in these patients (42). Innovative strategies such as point-of-care surveillance using cardiac biomarkers is an emerging tool to predict and monitor cancer therapy related cardiotoxicity. In a cross-sectional study, elevation in NT-proBNP was associated with reduced LVEF and pathological global longitudinal stress on echocardiogram (43). Further research is needed in this sphere to determine if biomarkers would allow the safe administration of these cancer therapies with less need for serial cardiac imaging.

The model of care in British Columbia, Canada, provides remote supervision of breast cancer care using local providers through the Community Oncology Network. Therefore, patients are linked with a specialist but are cared for locally (44). In systematic reviews, electronic consultations, termed “e-consults” are a promising tool with benefits such as good patient and provider satisfaction, delivery of a greater and timelier outpatient cardiac care, and savings from an economic standpoint (45, 46). The COVID-19 pandemic has led to the rapid integration and development of virtual care, likely reflecting the expedited policy changes around virtual care technologies (47). In a survey led by the Cardio-Oncology Collaborative Network, more then 85% of cardiologists and oncologists reported adopting telemedicine during the pandemic, the majority of which were in academic centers (48). While telehealth in oncology predates the pandemic, to our knowledge there is no published data describing telehealth in the sphere of cardio-oncology. The BREAST-AID study is currently evaluating the efficacy of a telemedicine cardio-oncological program for 200 patients in British Columbia (49).

### Real World Artificial Intelligence Applications in Medicine

Artificial intelligence (AI) has emerged as a highly supportive domain to enhance care delivery through telehealth tools. Ubiquitous in our daily lives, AI has been incorporated in medicine in the last decade to improve patient care and healthcare overall (50). Specific examples include wearable healthcare technology such as FitBits and smartwatches that analyze data for the users and their healthcare professional, EnsoSleep to help diagnose sleep disorders, Guardian Connect System to predict blood glucose changes, EchoMD for echocardiogram analysis, DermAssist an AI-powered tool to assist with diagnosis of dermatology conditions, and AI-powered rhythm analysis that offers automatic annotation and interpretation of ECG, to name of few.

In a recent retrospective study of 459 patients, AI-ECG was shown to be a powerful screening tool in assessing the risk of left ventricular dysfunction (LVD) among patients receiving anthracyclines or trastuzumab. Through a multivariable Cox-regression analysis, a positive AI-ECG independently predicted LVD at 5 years [HR: 2.12 (95% CI, 0.66–0.72); *p* < 0.0001] (51). Furthermore, a large-scale machine learning algorithm has the ability to integrate tremendous amounts of data to provide powerful cardiac risk stratification and predict cancer therapy-related cardiac dysfunction. While echocardiographic or laboratory test variables alone were predictive, the combination of both synergistically improved the performance of the model (52).

### Promising Solutions With Artificial Intelligence for Cardio-Oncology Care

AI is a promising avenue to enhance accessibility of cardio-oncology care and has supported telehealth through tele-assessment, tele-diagnosis, tele-interactions and tele-monitoring (53). For rural communities where laboratory tests might be less accessible, an AI-based blood diagnostics analyzer such as Sight OLO which is a compact, lightweight, electrically operated analyzer, might allow finger-prick sampling for laboratory tests in decentralized locations (54). This may facilitate the availability of NT-proBNP, a known prognostic and predictive biomarker in heart failure (55) which has emerging evidence in the context of patients treated with cardiotoxic cancer therapy (56). The European Society of Cardiology's 2016 expert consensus and European Society for Medical Oncology 2020 guidelines support baseline biomarker tests in anthracycline recipients (57). The European Society of Cardiology also recommends monitoring troponin to identify patients treated with high-dose chemotherapy who may benefit from an ACE-inhibitor (42). In terms of echocardiograms, drones built on AI are being developed to deliver healthcare equipment in remote areas (58) which may facilitate remote assessment of patients with cancer and survivors through virtual COCs. Artificial intelligence algorithms may also improve efficiency and reproducibility in echocardiogram measurements (59). EchoMD and AI-powered rhythm analysis, which are currently used, could be integrated to support interpretation of remote cardiac investigations. However, echocardiogram image acquisition is a user-dependent task owing to the nature of the modality therefore biomarkers may be an evolving way to monitor patients.

By leveraging photoplethysmography techniques, smartphone applications may be able to provide vital signs instead of requiring separate equipment, while remotely sharing the data with thehealth care provider (60). In cancer survivors, voice applications and analysis have shown promise in cardio-oncology, specifically promoting physical activity in cancer survivors through an in-home on-demand autonomous intelligent agent called MyCoach (61). Beyond the practical applications of AI in cardio-oncology, AI techniques have demonstrated the potential to markedly reduce the workload of health care providers (62).

## Proposed Cardio-Oncology Telehealth Model

To our knowledge, no organized models for telehealth cardio-oncology clinics exist worldwide. We propose the development and expansion of telehealth in cardio-oncology care in Canada and globally to serve both urban and rural patients.

In Canada, due to the nature of the health-care system, a provincial virtual COC with a pan-Canadian network, such as the Canadian Cardiac Oncology Network, could facilitate this collaboration. Although robust data is lacking on the proportion of patients living in rural settings that travel for care vs. those that move temporarily for cancer care, patients living farther from treating hospitals have worse prognosis attributed to several factors including difficulty with treatment compliance (63). In Australia, using telemedicine to facilitate nurse-supervised rural chemotherapy administration has been shown to significantly reduce the burden of travel for patients while providing safe delivery of cancer therapy locally (64). Therefore, telehealth in cardio-oncology for these patients could reduce financial and time-related barriers related to travel.

**Figure 1:**
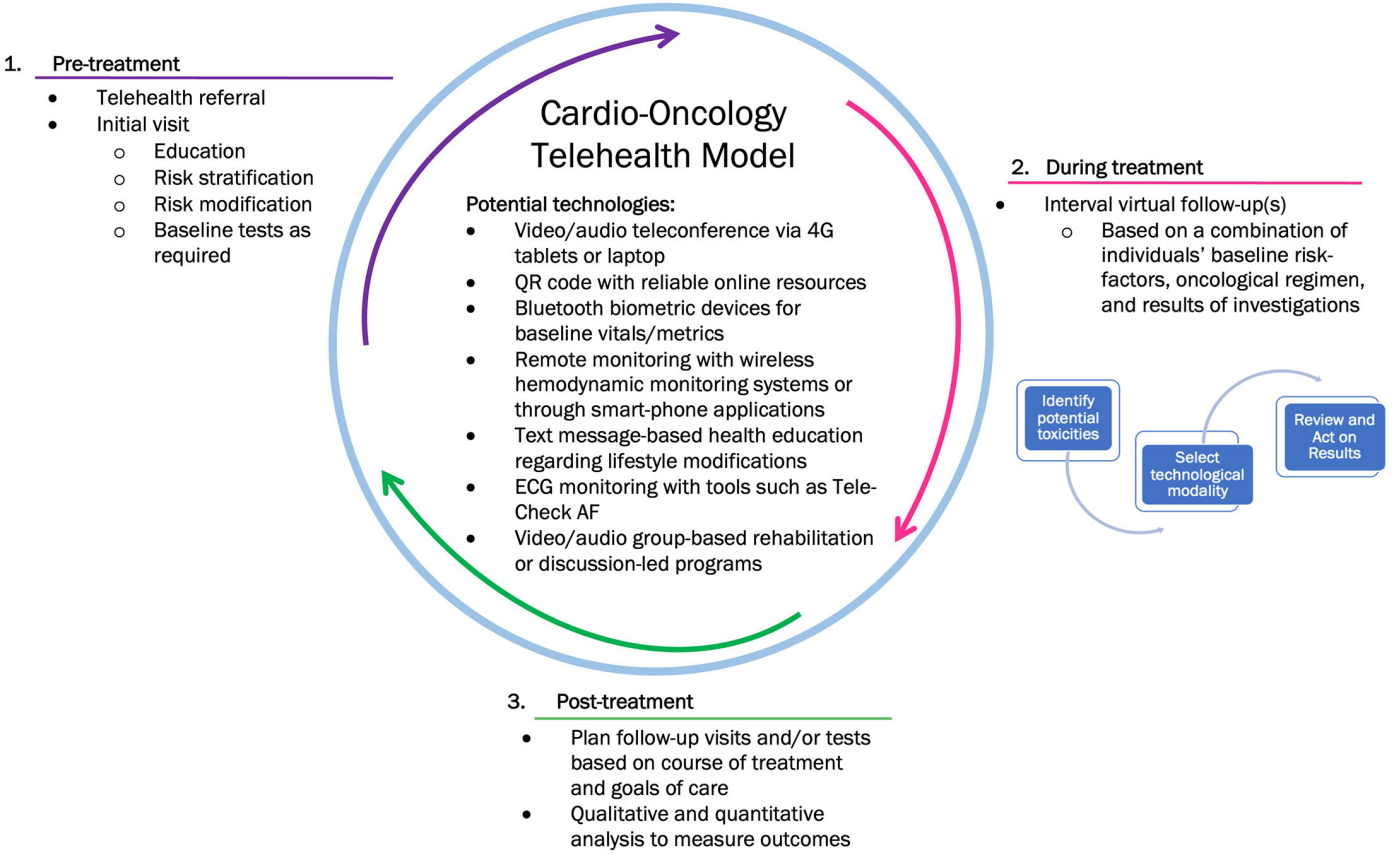
Proposed phases of care in a cardio-oncology telehealth model.

In the phases of care illustrated in Figure 1, we propose opportunities to communicate with and monitor patients remotely throughout their cancer treatment and survivorship. In the pre-treatment consultation, we propose an initial audio or audio/video conference with the patient. Numerous platforms have been developed including those embedded within electronic health records (EHR) such as In-Touch through EPIC and third-party platforms such as Zoom. Many software platforms prioritize being HIPAA compliant given the importance of cybersecurity and patient confidentiality (58). An initial multidisciplinary teleconference consultation allows the patient to be introduced to the members of the team and facilitates education, risk stratification, risk modification and baseline tests. Allied health professionals may be integrated; a nurse to provide education/awareness and gather baseline biometric data, a clinical pharmacist to perform a medication review and ensure ongoing compatibility of medications through all phases of treatment, and an administrative assistant to manage appointments and triage patient inquiries. Data could be gathered through a similar system as the PROTECT laboratory, currently used in the perioperative setting at the Population Health Research Institute (65).

During cancer treatments, there are several technologies to monitor and complement the patient history and symptomology. Table 1 outlines potential virtual surveillance strategies to personalize patient care based on their cancer therapy. For example, wireless vital sign monitoring to obtain blood pressure readings in patients on vascular endothelial growth factor signaling pathway inhibitors (66), CardioMEMs in patients with anthracycline-induced heart failure (67) and wearable devices such as a smartwatches or TeleCheck-AF systems to monitor arrhythmias in patients on tyrosine kinase inhibitors (68). During the COVID-19 pandemic, a de-novo virtual-hybrid cardio-oncology clinic integrated an element of remote patient-education through reliable internet resources accessible through QR codes (69). Studies have demonstrate that wireless implantable hemodynamic monitoring reduces rates of hospitalization in patients with heart failure over a 6 month follow-up (70) which may be useful in patients on anthracyclines or HER2-targeted therapies.

**Table 1:** Potential strategies for virtual surveillance based on cancer therapy.

**Cancer therapy**	**Virtual surveillance strategy**
**Conventional chemotherapies**	
Anthracyclines (e.g., doxorubicin, epirubicin)	Point-of-care troponin
Antimetabolites (e.g., 5-FU, capecitabine)	Remote cardiac ST segment monitoring
Alkylating agents (e.g., cyclophosphamide, melphalan) and Microtubule-binding agents (e.g., Paclitaxel)	Kardia or TeleCheck AF, virtual exercise rehabilitation for PAD such as MyCoach
Platinum-based therapy (e.g., cisplatin)	Automated BP machines
**Targeted agents**	
ALK inhibitors (e.g., alectinib, ceritinib, crizotinib)	Kardia or TeleCheck AF
BRAF inhibitors (e.g., dabrafenib) and MEK inhibitors (e.g., trametinib, binimetinib)	Point-of-care NT-proBNP
CDK4/CDK5 inhibitors	Kardia or TeleCheck AF
HER2 inhibitors	Point-of-care NT-proBNP
Ibrutinib	Kardia or TeleCheck AF
VEGF inhibitors	Automated BP machines
**Immunotherapy**	
Immune Checkpoint Inhibitors	Point-of-care troponin, remote ECG monitoring such as Eko Telehealth ECG live-stream, blood diagnostics analyzer such as Sight OLO

*5-FU, 5-fluorouacil; AF, atrial fibrillation; PAD, peripheral arterial disease; BP, blood pressure; ALK, anaplastic lymphoma kinase; BRAF, v-raf murine sarcoma viral oncogene homolog B1; MEK, mitogen-activated protein kinase kinase; CDK4/CDK5, Cyclin-dependent kinase 4/5; HER2, Human Epidermal Growth Factor Receptor 2; VEGF, Vascular endothelial growth factor*.

Many strategies from current virtual clinics of patients with CVD could be emulated in virtual COCs. For example, remote ECG monitoring, virtual cardiac rehabilitation, text message-based health education regarding lifestyle modifications, and group-based rehabilitation program (audio/video). The ONE TEAM study is currently investigating a remotely delivered, low touch, patient and primary care physician direction intervention in management of three CVD comorbidities including blood pressure measurements in patients with cancer (NCT04258813). In terms of frequency of visits, a triage algorithm similar to one suggested by Addison et al. could be considered; stratifying patients as high or low cardiac risk based on a combination of labs, biomarkers, historical data, cardiac remote monitoring and cancer therapy (71).

In the post-treatment phase, a shared-care model may be used to determine necessity and frequency of ongoing monitoring and follow-up based on potential long-term toxicities of oncological treatments received. In CVD patients, digital cardiac rehabilitation has been demonstrated to significantly reduce cardiovascular-related emergency visits and unnecessary rehospitalizations (72). In cardio-oncology care, exercise has been proposed in all phases as a viable non-pharmacological strategy to prevent, manage and improve cardiotoxicities (66). There may be an opportunity to offer remote exercise interventions post-cancer treatment (73) which may, in addition to providing physical benefit, also provide social support and connectedness that is likely limited in rural communities. Further research to understand its value and effectiveness is required. Implementing this model in a step-wedge randomized control trial may provide an opportunity to understand and evaluate the effects of this telehealth model.

## Conclusions

With increasing survivorship in cancer, addressing cardiovascular disease to mitigate morbidity and mortality is paramount in all phases of cancer patients' care. Cardio-oncology clinics currently exist mainly in urban cities worldwide including Canada. We propose a telehealth model of care integrating AI in cardio-oncology with the goal of addressing the urban-rural gap by improving access and highlighting the growing need for multidisciplinary clinics in this vital field.

## Author Contributions

CK contributed to the design of the tables and figures and writing of the manuscript. SD contributed to organization and supervision of the research. CK, MR-M, DL, and SD contributed to the literature review, writing of the manuscript and critical review. All authors contributed to the article and approved the submitted version.

## Conflict of Interest

SD declares Honoria from Astra Zeneca and Novartis. The remaining authors declare that the research was conducted in the absence of any commercial or financial relationships that could be construed as a potential conflict of interest.

## Publisher's Note

All claims expressed in this article are solely those of the authors and do not necessarily represent those of their affiliated organizations, or those of the publisher, the editors and the reviewers. Any product that may be evaluated in this article, or claim that may be made by its manufacturer, is not guaranteed or endorsed by the publisher.
